# A young woman presenting with acute knee pain: a case report

**DOI:** 10.4076/1757-1626-2-8212

**Published:** 2009-09-11

**Authors:** Yvonne Lo

**Affiliations:** 1 Department of Medicine, Family Medicine Unit, 3/F Ap Lei Chau Clinic, 161 Main Street, Ap Lei Chau, Hong Kong, China

## Abstract

A 35-year-old Chinese female experienced sudden onset of right knee pain one day after a charity hiking event with no history of injury. The pain was neither relieved with diclofenic sodium nor acetaminophen and she subsequently received various modalities of physiotherapy treatment. The pain disappeared within six weeks and she was able to resume full activity.

## Case presentation

I am a 35-year-old Chinese female family physician in Hong Kong and would like to share with you my story. I tore my left anterior cruciate ligament while playing hockey and had reconstruction surgery done more than ten years ago. The surgery was uneventful but unfortunately my knee became unstable again one year later which I attributed to poor rehabilitation on my part. Despite this, I continued with regular swimming practice although I never played hockey again. I developed an interest in hiking a few years ago and during the winter months I would go hiking every week for three to six hours. The instability in the left knee was not troublesome as long as I remained careful. Last year I climbed to the top of Mount Kinabalu (4095 m) and had no problem with my knees at all.

This year to challenge myself, I took part in a 50 km charity hiking event. It was the longest hike I had ever done, having completed the whole trail uneventfully within twelve hours. My knees felt fine until the following evening when the right knee, the 'good' one, started to hurt, particularly around the patella and yet there was no problem with my operated knee. There was no effusion and the range of movement was full. I thought it was patellar tendonitis, so I took diclofenic sodium 75 mg daily for five days. Yet the pain persisted and even got worse. I switched to acetaminophen 1 gm four times daily for two days, but again it did not help. The pain occurred not only when I walked on level ground but also while I was at rest. It even woke me up on a few nights. Eventually I visited a physiotherapist, twelve days after the hike. She examined my knee and it hurt when she compressed directly on my patella. I also had severe pain when she asked me to squat on my right leg. Her impression was patellofemoral syndrome and suggested acupuncture. Since the pain was absolutely terrible and it did not improve with medication, I decided to give it a try.

**Figure 1:**
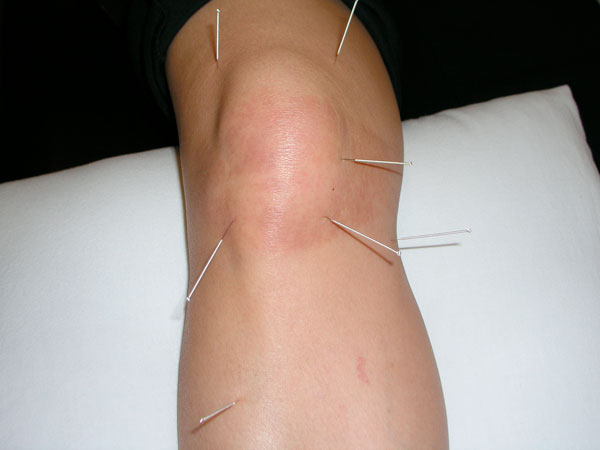
**Right knee with acupuncture needles**. Needles were inserted over seven trigger points around the right knee. Rash with sharp demarcation was a result of subsequent patella taping.

I had acupuncture treatment on three consecutive days and three times weekly thereafter. Ultrasound therapy was applied for five minutes prior to acupuncture. Seven needles were inserted around the knee (Figure 1) for thirty minutes and during which the physiotherapist would twirl the needles every ten minutes. Hot pack was then applied at the end for ten minutes. I was also instructed to do a series of stretching and muscle strengthening exercises at home every day for an hour. The progress was slow after six sessions, so patellar taping was applied. Unfortunately I developed a severe allergic reaction to the tape with pronounced redness, swelling and itch at the end of the day. The tape was removed and I continued the ultrasound therapy, acupuncture and had ice pack instead of hot pack. The pain and the rash gradually improved and by the fourth week of physiotherapy, I was able to resume full activity and my usual swimming practice with no pain at all. I had twelve acupuncture sessions altogether and the pain was completely gone within six weeks.

I reviewed the literature and found that patellofemoral syndrome is indeed quite a common condition although consensus has not yet been reached in regard to its aetiology and treatment [1]-[3]. Jensen et al. suggested that acupuncture may be an alternative treatment for patellofemoral syndrome [4]. Various reviews have tried to examine the efficacy of different forms of treatment, including exercise therapy, acupuncture, patellar taping, use of orthotics and braces and pharmacotherapy [5]-[11]. However further high quality, prospective, randomized, controlled, long term studies with validated outcome measures are still needed to develop treatment models for patellofemoral syndrome.

I must have overused and overloaded my right knee to compensate for the instability in my left knee although I was unlikely to be aware of that. I am not sure which modality helped me most, whether it was the ultrasound therapy, acupuncture, the hot or cold pack, the stretching exercise or the muscle strengthening exercise, but I am glad the pain has gone for two months now and I hope it will not recur.

## Consent

Written informed consent was not sought for publication of this case report since the author is the subject.

## Competing interests

The author declares that she has no competing interests.
